# SAS CARE 2 – a randomized study of CPAP in patients with obstructive sleep disordered breathing following ischemic stroke or transient ischemic attack

**DOI:** 10.1016/j.sleepx.2020.100027

**Published:** 2020-10-09

**Authors:** C. Bernasconi, S.R. Ott, F. Fanfulla, S. Miano, T. Horvath, A. Seiler, C.W. Cereda, A.-K. Brill, P. Young, L. Nobili, M. Manconi, C.L.A. Bassetti, SAS-CARE study group

**Affiliations:** a Department of Neurology, Inselspital, Bern University Hospital and University of Bern, Bern, Switzerland; b Department of Pulmonary Medicine, Inselspital, Bern University Hospital and University of Bern, Bern, Switzerland; c Sleep-Wake-Epilepsy Center, Department of Neurology, Inselspital, Bern University Hospital and University of Bern, Bern, Switzerland; d Pulmonary and Sleep Medicine, St. Claraspital, Basel, Switzerland; e Sleep Medicine, Neurocenter of the Southern Switzerland, Regional Hospital of Lugano, Lugano, Switzerland; f Faculty of Biomedical Sciences, Università della Svizzera Italiana, Lugano, Switzerland; g Stroke Center EOC, Neurocenter of the Southern Switzerland, Regional Hospital of Lugano, Lugano, Switzerland; h University Hospital Münster, Department of Neurology, Münster, Germany; i Department of Neurology, Ospedale Niguarda, Milano, Italy; j Sleep Medicine Unit, Istituti Clinici Scientifici Maugeri, Pavia, Italy; k DINOGMI, University of Genoa, Genoa, Italy; l Neurology Department, Sechenov First Moscow State Medical University, Moscow, Russia

**Keywords:** Stroke, CPAP, Randomized clinical trial, Clinical outcomes

## Abstract

**Objective/background:**

The benefit of Continuous Positive Airway Pressure (CPAP) treatment following ischemic stroke in patients with obstructive sleep-disordered breathing (SDB) is unclear. We set out to investigate this open question in a randomized controlled trial as part of the SAS-CARE study.

**Patients/methods.:**

Non-sleepy patients (ESS < 10) with ischemic stroke or transient ischemic attack (TIA) and obstructive SDB (AHI ≥ 20) 3 months post-stroke were randomized 1:1 to CPAP treatment (CPAP+) or standard care. Primary outcome was the occurrence of vascular events (TIA/stroke, myocardial infarction/revascularization, hospitalization for heart failure or unstable angina) or death within 24 months post-stroke. Secondary outcomes included Modified Rankin Scale (mRS) and Barthel Index.

**Results:**

Among 238 SAS-CARE patients 41 (17%) non-sleepy obstructive SDB patients were randomized to CPAP (n = 19) or standard care (n = 22). Most patients (80%) had stroke and were males (78%), mean age was 64 ± 7 years and mean NIHSS score 0.6 ± 1.0 (range: 0–5). The primary endpoint was met by one patient in the standard care arm (a new stroke). In an intent-to treat analysis disregarding adherence, this corresponds to an absolute risk difference of 4.5% or an NNT = 22. mRS and Barthel Index were stable and similar between arms. CPAP adherence was sufficient in 60% of evaluable patients at month 24.

**Conclusion:**

No benefit of CPAP started three months post-stroke was found in terms of new cardio- and cerebrovascular events over 2 years. This may be related to the small size of this study, the mild stoke severity, the exclusion of sleepy patients, the delayed start of treatment, and the overall low event rate.

## Highlights

• No benefit of CPAP started 3 months post-stroke was found.
• A sufficient CPAP compliance was observed over 2 years in 60% of patients.
• Studies of CPAP in mild stroke need to be large and include long-term outcomes.

## 1 Introduction

Sleep-disordered breathing (SDB) has a high prevalence and persistence over time after stroke or TIA (transient ischemic attack) [1] and has been recognized as a potentially modifiable risk factor for cardio- and cerebrovascular morbidity and mortality. In particular, obstructive sleep apnea (OSA) has been identified as an independent risk factor for acute cerebrovascular events. Both, OSA and central sleep apnea (CSA) are common in patients with incident stroke or TIA, but, as for the general population, OSA is the most frequently reported SDB pattern in the context of stroke.

Stroke has been reported to worsen a pre-existing SDB or even induce this condition [2,3]. Also, SDB, and its severity, has been associated with unfavorable stroke outcomes [[4], [5], [6], [7], [8], [9]]. Intermittent oxygen desaturations caused by OSA is assumed to be at the origin of oxidative stress and systemic inflammation, which in turn have been implicated in cardiovascular, metabolic as well as cognitive complications [10]. Possibly also due to the association with the presence of a patent foramen ovale, cardiac arrhythmias and hypercoagulability, OSA doubles the risk of stroke [11]. Regardless of epidemiological and experimental evidence, the value of SDB and OSA treatment by means of CPAP in patients with cardiovascular diseases remains controversial. Promising results were obtained in primary prevention. Longitudinal cohort studies pointed to a reduced risk of cerebral and heart ischemia [[12], [13], [14]] but randomized controlled evidence was less convincing [15]: a large study in OSA patients without daytime sleepiness failed to demonstrate a reduction in the incidence of hypertension or cardiovascular events except for about one third of patients with an adherence to CPAP >4 h/night. Findings from studies of secondary prevention were less clear. While some benefit appeared to be present in observational studies [16], according to a recent randomized study [17] and a meta-analysis [5] a significant benefit appears to be missing or only marginally significant for recurrent strokes in those patients who were adherent [17]. However, specifically in stroke, data from the literature suggest a more favorable effect as a recent meta-analysis of 10 randomized studies including less than 1000 patients [18]. However, samples were heterogenous, methodologies and outcomes were assessed differently and in all but 3 studies, randomized patients were followed up for 3 months or less.

A recent statement of the European Neurology, Pulmonology, Sleep, and Stroke Societies [19] has come to a similar conclusion, while emphasizing the existence of a bidirectional relationship between sleep and stroke and stressing the need for further mechanistic and therapeutic investigations.

Here we present results from SAS-CARE 2, a randomized study investigating the effect of CPAP started three months after stroke or TIA on post-event clinical outcomes over a period of two years. This project was part of SAS-CARE (Sleep Disordered Breathing in Transient Ischemic Attack (TIA)/Ischemic Stroke and Continuous Positive Airway Pressure (CPAP) Treatment Efficacy), a larger research initiative about sleep in stroke patients, which included in addition an observational study of patients who did not qualify for participation in the randomized study and a prospective polysomnographic investigation about the prevalence and 3-month evolution of SDB in stroke and TIA patients [20].

## 2 Material and methods

The design of the international SAS-CARE investigator-initiated study has been reported elsewhere [20]. The study (ClinicalTrials.gov identifier: NCT01097967) was performed in Switzerland (Bern, Lugano), Germany (Münster) and Italy (Milano). It was approved by the local ethics committees and conducted in accordance with the principles of good clinical practice and local regulations. The SAS-CARE-2 investigation was approved as part of the entire SAS-CARE project. It includes the randomized 2-parallel arm study presented in this report.

### 2.1 Participants

Between 29 September 2010 and 2 April 2014, we included patients who provided written informed consent, were aged between 35 and 75 years, had experienced an acute TIA or ischemic stroke requiring admission to a stroke unit within the last 60–90 days with appropriate diagnostic imaging (MRI or CT scan), and had an evaluable nocturnal polysomnography (PSG) at approximately 3 months after stroke. Exclusion criteria were: unstable clinical condition (cardio-respiratory or life-threatening medical conditions), current CPAP treatment or other SDB treatment during the last 3 months before stroke, non-ischemic events (intracerebral/subarachnoid hemorrhage), coma/stupor and any condition that may interfere with the acceptance of CPAP treatment. Randomization criteria additionally required patients to have moderate-to-severe OSA, defined as AHI ≥ 20 [21] in the PSG at 3 months post-stroke and to be non-sleepy as assessed by a score of <10 on the Epworth Sleepiness Scale (ESS). Patients not included in the randomized study were still included in the observational follow up and evaluated for the primary outcome. Those with moderate-to-severe SDB but who were sleepy were treated with CPAP within the clinical routine. The last patient left the study on 28 April 2016.

All PSG recordings (titanium; Embla Flaga, Reykjavik, Iceland) included six EEG channels, submental EMG, electro-oculogram, nasal airflow, 2 channels of breathing effort and oximetry, and EMG of both tibialis anterior muscles. PSGs after 3 months were recorded in the sleep laboratory between 10 pm and 8 am. All recordings were scored centrally and manually according to the AASM 2012 international criteria [22]. Hypopneas were scored when the peak signal excursions dropped by ≥30% of pre-event baseline for ≥10 s in association with either ≥3% arterial oxygen desaturation or an arousal. We did not differentiate between central and obstructive hypopneas. SDB was considered as OSA, if more than 50% of the apneas were of obstructive origin, while, in case of more than 50% of central apneas, SDB was classified as central.

### 2.2 Randomization and study treatment

Non-sleepy OSA patients with an AHI ≥ 20 in the PSG at 3 months post-stroke were randomized 1:1 to CPAP treatment (CPAP+ arm) or no CPAP treatment (CPAP− arm) without stratification, using a computer-generated randomization list produced by the coordinating center. In the CPAP arm, patients received an auto-CPAP device. The initial setting range of pressure was 7–14 cm H_2_O; afterward the pressure could be adapted according to local clinical practice to achieve optimal control of OSA.

### 2.3 Outcomes

Primary outcome was the occurrence of new vascular events (TIA/stroke, myocardial infarction/revascularization, hospitalization for heart failure, or unstable angina) or death (vascular or non-vascular) in the 24 months after stroke.

Secondary outcomes were the occurrence of the events defining the primary endpoint within 12 months post-event, Modified Rankin Scale (mRS) at 12 and 24 months and functional independence (mRS < 3) at 12 and 24 months.

Additional information collected in the study were location and etiology of stroke based on the criteria of the TOAST-study [23], stroke/TIA severity according to the National Institute of Health stroke scale (NIHSS) [24] on admission.

Adherence to CPAP treatment was assessed at each of the planned visits at 2–3 weeks, 4–6 weeks, 3–6, 12 and 24 months after randomization. Adherence was classified as good if the device was used for ≥5 h per night in at least 70% of the nights. Sufficient adherence was defined for a use of CPAP for at least 4 h per night during at least 70% of the nights. Insufficient adherence was defined as CPAP use <4 h per night or less than 70% of nights.

### 2.4 Statistical analysis

The sample size calculation assumed event rates at 2 years of 10% and 20% in the CPAP+ and CPAP− arms, respectively. A total of 220 patients were expected to be randomized to provide a power of 80% for a two-sided test at the 5% significance level. Recruitment was prematurely discontinued in April 2014 after an assessment performed by the study management team indicated that the overall event rate was clearly lower than anticipated, and that the study would have required an extension clearly beyond the planned time frame.

The primary analysis was based on the intent-to-treat population, including all randomized patients allocated to treatment arms according to randomization. Missing values were not imputed and sensitivity analyses were performed with different imputation rules. Fisher's exact test was used to compare groups for categorical variables and the Wilcoxon rank-sum test for continuous variables and Wilcoxon signed-rank test. All statistical tests were two sided and conducted at the 5% significance level without adjustment for multiplicity.

## 3 Results

### 3.1 Patient disposition and baseline characteristics

**Fig. 1:**
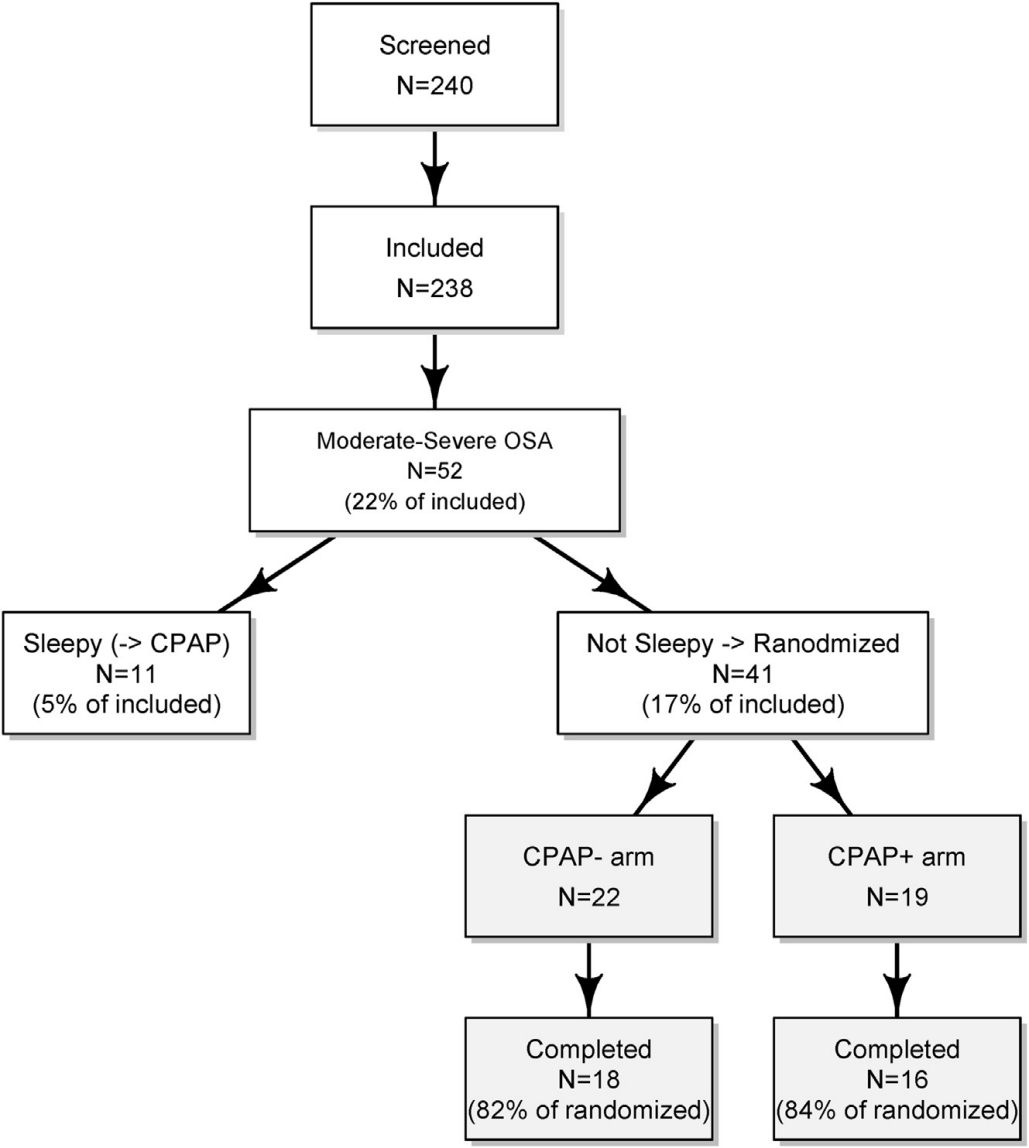
Patient disposition.

As displayed in Fig. 1, out of 240 patients screened for the different parts of the SAS-CARE study, 238 were included in the observational follow up (one did not provide informed consent and one did not have an evaluable PSG). Of those patients, 52 (22%) displayed moderate-to-severe OSA in the PSG three months after stroke, and 41 (17%) were non-sleepy and therefore eligible for randomization into the treatment or control arms of SAS-CARE 2. All received the intended treatment and were analyzed according to the treatment arm defined by randomization. Most patients were randomized in one of the two main study centers Bern and Lugano, providing 49% and 39% of randomized patients, respectively. Study completion rates were 82% and 84% in CPAP− and CPAP+, respectively, with seven patients (CPAP− n = 4, CPAP+ n = 3) being lost to follow-up.

Non-randomized patients (n = 197, 83%), who had either AHI < 20 or were sleepy, were also followed up in parallel to the randomized cohort. Sleepy patients with at least moderate OSA (n = 11, 5%) were treated with CPAP as per clinical practice.

Key background characteristics of randomized patients (ITT population), including clinical presentation and treatment, are shown in Table 1.

**Table 1:** Baseline characteristics of randomized patients with sleep-disordered breathing.

	CPAP− N = 22	CPAP+ N = 19	All N = 41
TIA	3 (13.6%)	5 (26.3%)	8 (19.5%)
Clinical infarct type			
No information	0 (0%)	4 (21.1%)	4 (9.8%)
LACI	4 (18.2%)	3 (15.8%)	7 (17.1%)
TACI	1 (4.5%)	1 (5.3%)	2 (4.9%)
PACI	8 (36.4%)	4 (21.1%)	12 (29.3%)
POCI	9 (40.9%)	7 (36.8%)	16 (39%)
Stroke etiology (TOAST)			
No information	0 (0%)	1 (5.3%)	1 (2.4%)
Large artery	3 (13.6%)	1 (5.3%)	4 (9.8%)
Cardioembolic	7 (31.8%)	3 (15.8%)	10 (24.4%)
Small artery	5 (22.7%)	2 (10.5%)	7 (17.1%)
Dissection or other	1 (4.5%)	2 (10.5%)	3 (7.3%)
Unknown – evaluated	3 (13.6%)	5 (26.3%)	8 (19.5%)
Unknown – in evaluation	1 (4.5%)	1 (5.3%)	2 (4.9%)
>one	0 (0%)	1 (5.3%)	1 (2.4%)
Patent foramen ovale only	1 (4.5%)	2 (10.5%)	3 (7.3%)
Atherosclerosis < 50%	1 (4.5%)	0 (0%)	1 (2.4%)
Aorta plaque	0 (0%)	1 (5.3%)	1 (2.4%)
Thrombolysis	4 (18.2%)	2 (10.5%)	6 (14.6%)
Sex – male	17 (77.3%)	15 (78.9%)	32 (78%)
Age [years]	64.7 ± 6.6/63 (59; 76)	64.1 ± 6.7/63.5 (60; 69)	64.4 ± 6.6/63 (59; 69)
Body mass index [kg/m^2^]	27.5 ± 5.6/26.0 (23.1; 29.3)	28.9 ± 5.1/27.8 (25.0; 31.7)	28.0 ± 5.2/26.2 (23.7; 30.7)
Modified Rankin scale (numeric)	0.62 ± 0.80/0 (0; 1)	0.79 ± 0.63/1 (0; 1)	0.70 ± 0.72/1 (0; 1)
NIHSS total score	0.48 ± 1.12/0 (0; 1)	0.63 ± 0.96/0 (0; 1)	0.55 ± 1.04/0 (0; 1)
Barthel index	99.8 ± 1.1/100 (100; 100)	99.7 ± 1.2/100 (100; 100)	99.7 ± 1.2/100 (100; 100)
Essen stroke risk score	2.38 ± 1.36/2 (1; 4)	2.28 ± 1.27/2 (1.25; 3.75)	2.33 ± 1.3/2 (1; 4)
AHI [events/h]	32.0 ± 11.3/26.9 (23.0; 37.8)	36.7 ± 14.8/32.9 (25.0; 42.8)	34.3 ± 13.2/32.3 (23.5; 42.2)
mean O_2_ saturation [%]	92.4 ± 2.1/92.9 (90.3; 94.1)	92.7 ± 1.5/92.9 (92.2; 93.4)	92.6 ± 1.8/92.9 (91.7; 93.7)
Minimum O_2_ saturation [%]	80.7 ± 6.1/82.0 (76.0; 85.0)	82.1 ± 3.1/82.0 (80.0; 84.5)	81.3 ± 4.9/82.0 (79.0; 85.0)
Saturation Time <90% [min]	78.5 ± 99.7/24.2 (4.9; 156.2)	22.2 ± 33.7/8.6 (6.0; 25.0)	53.9 ± 82.1/14.9 (5.1; 48.7)

For categorical variables data are presented n (% of N in the group), for continuous variables as mean ± standard deviation/median (first quartile; third quartile). For variables not reflecting characteristics of the stroke (type, etiology, treatment) baseline refer to the randomization visit. Abbreviations not defined elsewhere: LACI: lacunar infarct; TACI: total anterior circulation infarct; PACI: partial anterior circulation infarct; POCI: posterior circulation infarct; TOAST: Trial of Org 10172 in acute stroke treatment.

Most patients (74%) were included following an ischemic stroke, and a slightly larger number of TIA patients was assigned to CPAP (3 vs. 5, resulting in an almost double proportion). Otherwise, baseline characteristics appeared well balanced between the two treatment arms. In general, events were of rather modest severity even in the subset of patients with a stroke at study entry (mean mRS in stroke patients 0.73 ± 0.75 and mean NIHSS 0.65 ± 1.12).

Compared to randomized patients, the observational non-randomized cohort had slight differences: patients were more often female (28%), younger (mean age 60.8 ± 9.5 years) had less frequently a TIA (13.1%), and a more severe stroke (NIHSS 0.86 ± 1.58, mRS 0.79 ± 0.87, respectively) and similar BMI (27.3 ± 4.6). Their Essen stroke risk score (1.9 ± 1.3) was lower. The mean AHI was 11.4 ± 13.2/h, and 37.9 ± 15.2/h for the small group of sleepy patients with moderate to severe SDB.

### 3.2 Primary endpoint

The key results are presented in Table 2. Within the study period of 18 months following randomization (2 years after the stroke) one new event was registered in the randomized patients: one recurrent ischemic stroke was reported at the month 12 visit in a 71-year-old female patient from the CPAP− arm who had initially suffered a large artery stroke. The overall event rate (95% Clopper–Pearson CI) was 2.4% (0.1%–12.9%), and the event rate in the CPAP− arm was 4.5% (0.1%–22.8%). No death occurred. No statistically significant between-arm difference (p > 0.7 in all tests) was detected, neither in the primary analysis that did not impute missing outcome data, nor in supportive analysis where patients with unknown outcome were either evaluated as a separate category or considered as having met the primary endpoint (event rates 22.7% and 15.8% in CPAP− and CPAP+, respectively, risk ratio with 95% CI: 0.695 [0.191, 2.532]).

**Table 2:** Primary endpoint for randomized non-sleepy patients with sleep-disordered breathing (death or new vascular event).

	CPAP− N = 22	CPAP+ N = 19	All randomized N = 41
Yes	1 (4.5%)	0 (0%)	1 (2.4%)
No	17 (77.3%)	16 (84.2%)	33 (80.5%)
Unknown	4 (18.2%)	3 (15.8%)	7 (17.1%)

Among 150 patients from the non-randomized cohort who were evaluable for the primary endpoint, the event rate was 10.6% (16 events). In 7 of the sleepy patients who exhibited moderate to severe OSA, received CPAP treatment and were evaluable for the primary endpoint (at inclusion there were 11 such patients), the event rate was 28.6% (2 events).

### 3.3 Secondary endpoints

**Fig. 2:**
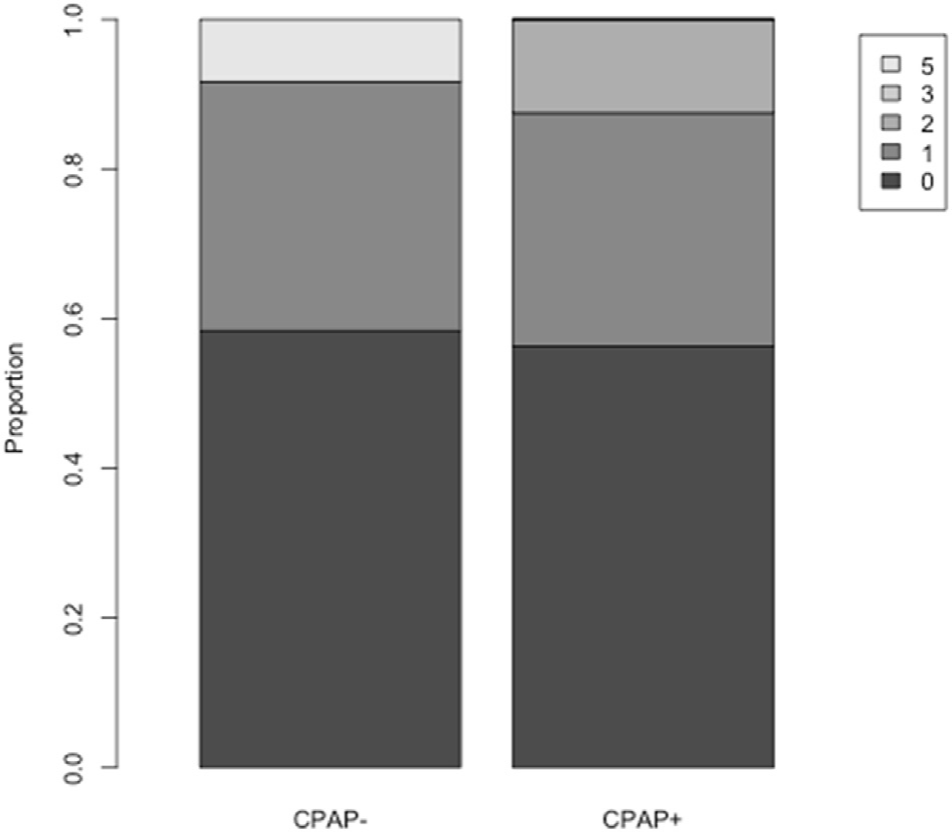
mRS at month 24.

The level of disability among randomized patients, as measured by the mRS and the Barthel index at month 12 and 24 (Table 3) was in general rather low and not different between the two treatment arms (mRS: p > 0.5 for all comparisons) for both timepoints. Fig. 2 shows the mRS as a categorical variable. The patient who met the primary endpoint was the only one with a mRS>2 among the randomized patients.

**Table 3:** Secondary efficacy endpoints at month 12 and 24.

		CPAP−	CPAP+	All randomized
Month 12				
Modified Rankin scale (numeric)	N (evaluable patients)	16	17	33
	mean ± standard deviation	0.50 ± 0.63	0.53 ± 0.72	0.52 ± 0.67
	median (first quartile; third quartile)	0 (0; 1)	0 (0; 1)	0 (0; 1)
	Min; max	0; 2	0; 2	0; 2
Barthelindex	N (evaluable patients)	16	17	33
	mean ± standard deviation	99.4 ± 1.7	98.2 ± 5.0	98.8 ± 3.8
	median (first quartile; third quartile)	100 (100; 100)	100 (100; 100)	100 (100; 100)
	Min; max	95; 100	80; 100	80; 100
Month 24				
Modified Rankin scale (numeric)	n (evaluable patients)	12	16	28
	mean ± standard deviation	0.75 ± 1.4	0.56 ± 0.72	0.64 ± 1.06
	median (first quartile; third quartile)	0 (0; 1)	0 (0; 1)	0 (0; 1)
	Min; max	0; 5	0; 2	0; 5
Barthel index	n (evaluable patients)	12	15	27
	mean ± standard deviation	94.2 ± 20.2	99.0 ± 2.8	96.9 ± 13.5
	median (first quartile; third quartile)	100 (100; 100)	100 (100; 100)	100 (100; 100)
	Min; max	30; 100	90; 100	30; 100

In the cohort of non-randomized patients, the mean mRS at week was minimally lower (0.60 ± 0.74).

### 3.4 Adherence to CPAP treatment

As summarized in Table 4, among patients who were evaluated, good adherence was the most common reported category (50%–79% of patients). However, it has to be noted that an assessment was not performed in a relevant proportion of CPAP+ patients: at the initial assessment (Week 3–4) 80% of CPAP+ patients were evaluable for adherence and this proportion progressively decreased to 53% at month 24.

**Table 4:** Adherence to CPAP treatment over time in the CPAP+ arm.

	All CPAP+ patients (N = 19)		CPAP+ patients assessed for adherence		
	Assessed	Not assessed	Insufficient	Sufficient	Good
Week 3–4	15 (78.9%)	4 (21.1%)	6 (40.0%)	1 (6.7%)	8 (53.3%)
Month 3	14 (73.7%)	5 (26.3%)	3 (21.4%)	1 (7.1%)	10 (71.4%)
Month 6	14 (73.7%)	4 (26.3%)	2 (14.3%)	1 (7.1%)	11 (78.6%)
Month 12	12 (63.2%)	7 (36.8%)	2 (16.7%)	3 (25%)	7 (58.3%)
Month 24	10 (52.6%)	9 (47.4%)	1 (10%)	3 (30%)	6 (60%)

## 4 Discussion

The key observation from our study is that the overall rate of new cardio or cerebrovascular events in the 2 years following the initial ischemic cerebrovascular event was very small: only one event was reported with an overall event rate of 2.4% among randomized patients and 3.0% among those who were evaluable. The overall disability level was also low.

Given that the observation time was rather long and that the key endpoints were of clinical nature, a potential clinical implication of a low event rate is that CPAP treatment may not be useful for secondary cardiovascular prevention in non-sleepy patients with very mild strokes or TIA if started three months after stroke/TIA. However, we cannot exclude potential benefits on an even longer time scale. Given the small sample size and small variability in our sample, our ability to make predictions beyond the study horizon by means of statistical modeling is restricted. In addition, we cannot exclude benefits in clinical domains we did not explicitly assess as eg improvement of cognitive function [25]. For future trials, it will be important to evaluate the feasibility of identifying a sufficient number of patients with severe residual symptoms and high recurrence risk who would be still willing to participate in a clinical investigation of this kind.

The low event rate and mild disability level can be in part attributable to the predominantly mild stroke severity and the young age in our patient population. On the other hand, in 150 patients who were observed outside the randomized study and evaluable for the primary endpoint, the event rate was larger (10.6%). Of note, in a small subgroup of these patients who exhibited a moderate to severe SDB but were sleepy and therefore assigned to CPAP treatment, two events were observed (event rate 28.6%). The higher event rate in non-randomized patients, who had roughly the same risk profile as randomized patients except for SDB and sleepiness, casts doubts on the representativeness of the findings of the randomized study. Also, it constitutes a severe limiting factor when it comes to the interpretation of the negative study results in terms of effect size for the intervention under investigation (no evidence for a difference between the two treatment arms neither for primary nor for secondary outcomes). Among prospective investigations with a design comparable to ours and a sufficient follow-up for clinical events, a recent study [26] reported an overall event rate of 10% at 12 months in 70 patients, who were, however, in clearly worse conditions at baseline (mean mRS 1.95). Similar statements regarding baseline characteristics and outcomes can be made for studies of CPAP for stroke or TIA patients with obstructive sleep apnea [27]. In a randomized study of nasal CPAP, that also included patients with a clear higher mean mRS (approximately 2.5) the event rate at 2 years was approximately 10% [28]. The apparent discrepancy in the key findings among patient groups may be in relation with the small sample size of our study: the statistical precision of the computed event rates is in fact small, with the upper limit of the 95%-CI exceeding 20% for primary endpoint in the CPAP− arm. Stated differently, given the overall low observed event rates, the recruited sample would have provided a very low statistical power to detect even rather large effects in the randomized study, for instance <20% power for event rates of 5% vs. 20%. The level of certainty of our findings is therefore rather small. Based on the primary event rate, assignment to CPAP in sleepy SDB patients, despite the absence of a proper control group, did also not appear to be beneficial.

Additional limitations need to be mentioned. Firstly, there were some missing data for the primary and secondary endpoints mainly due to loss to follow-up. However, despite being slightly more frequent in CPAP− patients, loss to follow-up did not substantially affect the significance of the treatment effect. Also, congestive heart failure was not monitored systematically by echocardiography, preventing an assessment of the impact of CPAP on central respiratory effects. It also has to be considered that CPAP use does not necessarily reflect a sufficient control of OSA. We aimed to control SDB but cannot corroborate this with objective data, such as residual AHI. Finally, the study was conducted in central Europe, with a limited diversity in the patient population.

Although largely incomplete, as for several other similar studies [18], the assessment of adherence indicated that CPAP may be acceptable to a large proportion of patients. Because no clinical events were reported in CPAP+ patients we were unable to evaluate the role of adherence with respect to the CPAP effect on stroke outcomes.

## 5 Conclusion

Our results do not provide support to the use of CPAP for the secondary prevention of cardio- or cerebrovascular events over a period of two years in non-sleepy SDB patients following a mild stroke if CPAP is started 3 months after the event. Larger prospective controlled trials including more severe strokes are necessary to address the effects of early SDB treatment and the need of SDB re-evaluation in the chronic stroke phase in specific subgroups, eg patients with predominant CSA.

## Conflict of interest

None declared.

## CRediT authorship contribution statement

**C. Bernasconi:** Formal analysis, Writing - original draft, Writing - review & editing. **S.R. Ott:** Investigation, Writing - review & editing. **F. Fanfulla:** Investigation, Writing - review & editing. **S. Miano:** Investigation, Data curation, Writing - review & editing. **T. Horvath:** Investigation, Writing - review & editing. **A. Seiler:** Investigation, Writing - review & editing. **C.W. Cereda:** Investigation, Writing - review & editing. **A.-K. Brill:** Investigation, Writing - review & editing. **P. Young:** Investigation, Writing - review & editing. **L. Nobili:** Investigation, Writing - review & editing. **M. Manconi:** Investigation, Writing - review & editing. **C.L.A. Bassetti:** Conceptualization, Supervision, Funding acquisition, Writing - review & editing.
